# Prevalence of *Eucoleus garfiai* in Wild Boars Hunted at Different Altitudes in the Campania and Latium Regions (Italy)

**DOI:** 10.3390/ani13040706

**Published:** 2023-02-17

**Authors:** Karen Power, Manuela Martano, Nadia Piscopo, Paolo Viola, Gennaro Altamura, Vincenzo Veneziano, Ana Carvajal Urueña, Luigi Esposito

**Affiliations:** 1 Department of Veterinary Medicine and Animal Production, University of Napoli—Federico II, 80137 Naples, Italy; 2 Department of Agriculture and Forestry Science, University of Tuscia, 01100 Viterbo, Italy; 3 Department of Animal Health, University of León, 24071 Leon, Spain

**Keywords:** bioindicator, histopathology, nematode, soil quality, wild boar

## Abstract

**Simple Summary:**

Recent findings of the nematode *Eucoleus garfiai* in wild boars across different countries, and more lately in southern Italy, have brought up the need for collecting epidemiological data on this parasite. In the present study, the prevalence of *E. garfiai* was analyzed in relation to altitude in different provinces of the Campania and Latium regions located, respectively, in southern and central Italy. Results showed that the parasite is more often found at altitudes higher than 900 m above sea level. Some species of earthworms are intermediate hosts of *E. garfiai* and it is well known that earthworms are more present in high quality soils, which are more likely found at high altitudes where anthropogenic interventions are less frequent. Therefore, we can suggest that the higher prevalence of *E. garfiai* above 900 m above sea level is probably linked to a higher presence of earthworms in the soil, due to its higher quality in these areas.

**Abstract:**

Recent reports of *Eucoleus garfiai* in wild boars in southern Italy have highlighted the need for collecting epidemiological data on the presence of this parasite and understanding the role of possible interactions between wild boars, *E. garfiai*, and the environment. This study analyses, using histopathological and biomolecular techniques, the presence of *E. garfiai* in tongue samples of wild boars hunted in four provinces of the Campania and Latium regions (Italy), in areas located above and below 900 m above sea level (asl). Histopathological examinations revealed the presence of adults and eggs of nematodes, which were subsequently identified as *E. garfiai* by biomolecular analysis, in the tongue epithelium. The detection of the parasite was more frequent in samples collected from hunting areas located above 900 m asl than in those collected from areas located below 900 m asl (66.67% vs. 38.09%; *p* < 0.01). Some species of earthworms are intermediate hosts of *E. garfiai* and it is well known that earthworms are more present in high quality soils. Therefore, we can suggest that the higher prevalence of *E. garfiai* at higher altitudes is probably linked to a greater presence of earthworms in the soil, due to its higher quality in these areas.

## 1. Introduction

The lives of wild animals are strictly connected to the environment in which they live and to the other living beings with whom they share it [1,2,3]. The strong relationship between animals and the environment makes the contact of wild species with numerous saprophytic and/or pathogenic organisms particularly easy, especially with those living in waters and soil [4,5]. The modern approach to One Health obliges researchers to pay attention to the different environments that host biodiversity. Living beings are, in fact, conditioned by the size of living spaces, the availability of trophic resources, pathogens and anthropic pressures. In the case of large wild mammals, some studies have demonstrated relationships between oxidative stress markers and living environments both for individual populations and for the interspecific territory sharing [6,7]. Great interest nowadays is given to soil born-diseases [8,9], and, in wildlife, studies are mainly focused on soil pathogens which are of public health interest such as *Francisella tularensis*, *Yersinia pestis*, *Bacillus anthracis*, *Coxiella burnetii*, Avian influenza virus H5N1 [10], Swine flu H1N1 [11], Coronavirus [12], and different helminths [13] as they can negatively affect humans, livestock and domestic animals [14,15,16,17,18,19]. However, more and more studies have underlined the importance of pathogenic and saprophytic microorganisms and macrorganisms, such as earthworms, as a valuable tool for a variety of ecology-based applications, such as environmental bioindicators [20,21,22].

Wild boars (*Sus scrofa*) are extensively distributed worldwide and, starting from the mid-twentieth century, a significant increase in their population in Europe and Italy has been described with a consequent enlargement of their habitat [23,24,25] even though a reliable estimate of the number of individuals present is not known (unpublished data). As omnivores, wild boars can feed on a wide range of foods of plant origin (roots, rhizomes, crops) and of animal origin (insects, birds, snails), according to the availability of food sources [26,27]. Among the animal sources found in the stomachs of hunted wild boars, earthworms appear to be a significant component probably due to their availability in the first layers of soil, to their great energy and nutrient content, and to the rooting feeding habits of wild boars [28,29,30]. Nevertheless, ingestion of earthworms can also represent an important source of parasitic infections as these invertebrates are often intermediate hosts of nematodes which can parasitize the lungs of wild boars [31,32,33,34].

Recently, the nematode *Eucoleus garfiai*, synonym *Capillaria garfiai*, was detected in adult wild boars hunted in southern Italy [35]. Previous studies had already described the presence of *E. garfiai* in domestic and wild swine in Spain [36], Austria [37], Japan [38], central Italy [39] and Iran [40]. Adults and eggs can be observed in the tongue of wild boars after ingestion of infected earthworms, such as *Lumbricus terrestris*, *Allolobophora caliginosa* and *Allolobophora rosea*, which act as intermediate hosts [41]. Histopathological examination of infected tongues reveals moderate inflammation of the tissue characterized by infiltration of lymphocytes, plasma cells and eosinophils, suggesting reduced pathogenicity of the parasite [38]. Although *E. garfiai* can be considered a non-zoonotic and slightly pathogenetic parasite, data on the presence of this nematode in wild boars should be increased. Moreover, intraspecific relationships between wild boars–earthworms–environment should be explored further to better understand their epidemiological and ecological relevance. Therefore, considering the novelty of the presence in southern Italy and the lack of information about *E. garfiai*, the aim of this study was to assess by histopathological and biomolecular techniques the presence of *E. garfiai* in wild boars in different areas of the Campania and Latium regions (Italy) located at different altitudes.

## 2. Materials and Methods

**Figure 1:**
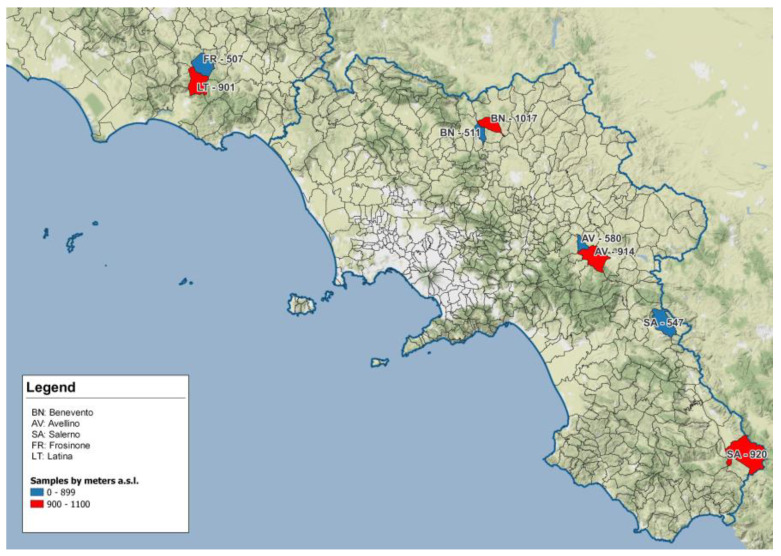
Geographical map presenting localization of sampling sites in Campania and Latium region: sites located >900 m asl are in red, sites located <900 m asl are in blue. Blue line indicates the geographical border between regions.

A total of 69 wild boars were collected in 8 different areas pertaining to 4 different provinces of 2 regions of southern and central Italy (3 in Campania and 1 in Latium) during the months of October, November and December of the 2021–2022 hunting season. Sampling locations are shown in Table 1 and Table 2 and identified on the map in Figure 1. Areas were selected according to altitude and to the presence of wild boar hunting teams which could ensure sample availability.

Three samples (1 from CF AV < 900 m and 2 from PL BN > 900 m) were excluded as they were in a bad state of conservation; therefore, 66 wild boar tongue samples were isolated and transported by members of the laboratory of Animal Husbandry of the Department of Veterinary Medicine and Animal Productions, University of Naples “Federico II” in containers filled with 10% buffered formalin to the laboratory of Veterinary General Pathology and Anatomical Pathology of the Department of Veterinary Medicine and Animal Productions for further histopathological processing. Samples of 2 cm width were cut from each tongue and routinely processed for histopathological examination as previously described [42]. Briefly, they were fixed in 10% buffered formalin, paraffin-embedded, stained with haematoxylin and eosin (HE). After, tissue preparations were observed by light microscopy (Microscope Nikon Eclipse E-600, Tokyo, Japan) to identify the possible presence of parasites and morphological alterations. Parasite egg size was measured using a free image analysis software (Image J). Two samples were also stored at −20 °C and two adult parasites (one for each sample) were isolated under a stereomicroscope and stored at −20 °C for biomolecular analysis. DNA extraction and Polymerase chain reaction (PCR were performed as previously described [43,44]. The following primer pair: *E. garfiai* 18s_FW: 5′-GTCGTCGTCGAGATGAGTCG-3′, *E. garfiai* 18s_REV: 5′-TCTCTCCGGAATCGAACCCT-3′ (annealing T 60 °C), designed on the sequence available on GenBank (MW947272.1), was employed for amplification of a specific fragment (180 bp) of 18s ribosomal RNA gene of *E. garfiai.* A positive control mimicking *E. garfiai* 18s intended amplicon was synthesized according to gBlocks Gene Fragments technology (Integrated DNA Technologies Coralville, IA, USA) and run along with PCR reactions. Moreover, one no template control (NTC) was included as negative control. Amplification products were then migrated by electrophoresis on 2.5% agarose gel in TAE buffer (Tris-Acetate-EDTA) along with a 100 bp molecular marker (Bioline), stained with ethidium bromide and observed under UV with the ChemiDoc gel scanner (Bio-Rad). One representative PCR product was purified using the QIAquick PCR Purification Kit (Qiagen, Hilden, Germany) according to the manufacturer’s protocol, and submitted for sequencing at BMR Genomics (Padova, Italy). The obtained sequence was aligned with available sequences of *E. garfiai* from GenBank.

In order to evaluate the possible relationship between the presence/absence of the parasite in wild boars and altitude (below and above 900 m asl), comparison of the percentages was carried out with the Chi-squared test of independence using the JMP^®^ PRO 14 software.

## 3. Results

**Figure 2:**
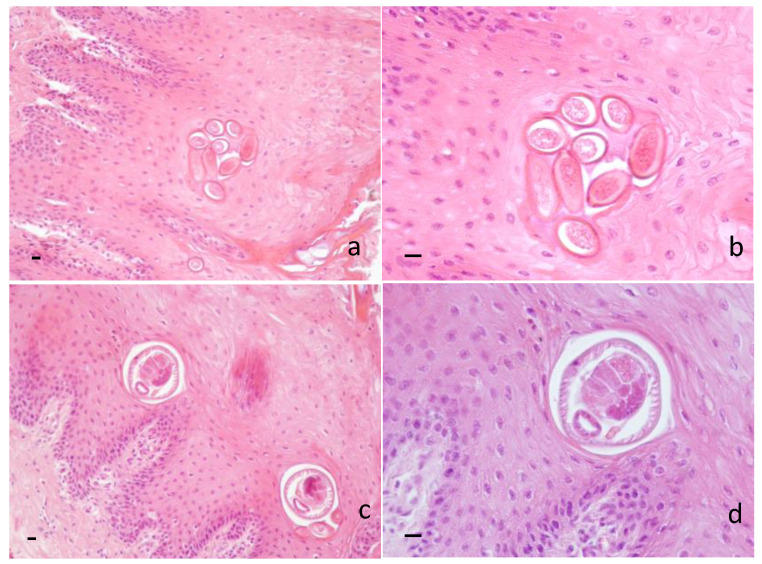
Wild boar. Histological section of tongue. (**a**) Barrel-shaped eggs of *E. garfiai* showing protruding polar plugs in the prickle layer of the epithelium. 20 × HE. Scale bar 10 µm; (**b**) eggs of *E. garfiai* 40 × HE. Scale bar 10 µm; (**c**) transversal section of adults of *E. garfiai* in the prickle layer of the epithelium. 20 × HE. Scale bar 10 µm; (**d**) transversal section of adult of *E. garfiai* 40 × HE. Scale bar 10 µm.

Histopathological examination revealed the presence of several sections of adults and/or eggs of helminths in the dorsal lingual epithelium of 32/66 samples (48.48%) (for a comprehensive view of results, see Table S1). Barrel-shaped eggs, showing two protruding polar plugs and measuring approximately 50 µm in the longitudinal direction, were observed throughout the corneal layer and the prickle layer (Figure 2a,b), while fragments of *E. garfiai* adults and/or whole adults, measuring approximately 70 µm in the transversal direction, were identified in the prickle layer, often in proximity to the basal layer (Figure 2c,d). Histopathological examination revealed hyperkeratosis, parakeratosis and strong exfoliation of the corneal layer of the lingual epithelium; few lymphocytes, plasma cells and eosinophils were noticed below the basal cell layer. Histological localization of adults and eggs and microscopic morphological appearance suggested identification of the parasite.

A fragment of the expected size (180 bp) of *E. garfiai* was successfully amplified from both the analyzed samples and, as expected, from the positive control but not in NTC. Sequence analysis confirmed the identity of the amplicon, in agreement with the histopathological identification of the parasites. Statistical analysis highlighted that samples collected from hunting areas located above 900 m asl presented a higher prevalence of the parasite compared to those collected from areas located below 900 m asl (66.67% vs. 38.09%; *p* < 0.01) (Table 3). This result was consistent in all areas (Table 3).

## 4. Discussion

The growth in population of wild ungulates and the expansion of their living habitat towards more anthropogenic areas pose numerous concerns under an ecological and sanitary point of view. Increased use of lands for agricultural purposes, deforestation and inhabitation of suburban areas have modified the extension of areas inhabited by humans and wild boars, causing overlapping between the two populations, and developing more chances of contact exposure among wild boars and humans and livestock [45]. Wild boars can harbor many infectious agents which are transmissible to livestock, domestic animals and humans, and parasites represent a noteworthy category [46,47]. However, not all parasites infecting wild boars are zoonotic and pathogenic. As previously reported, *E. garfiai* is a non-zoonotic non-pathogenic helminth which can be found in the tongue of wild boars without causing severe lesions. Histopathological results of our study confirmed the presence of eggs and adults in tongue samples associated with mild inflammatory alterations, as previously described [35,38]. Recent findings of *E*. *garfiai* in southern Italy have raised interest in collecting more data to better understand the ecology and epidemiology of this particular parasite. In this study, we investigated the prevalence of *E. garfiai* in different areas located below and above 900 m asl and the results showed a higher prevalence of *E. garfiai* in sampling areas located above 900 m asl. Altitude has frequently been used as a parameter for studying biodiversity richness and it was previously reported that earthworms’ communities adapt well to different gradients of altitude, increasing in number and variety of population, probably due to lower anthropogenic influence and higher soil quality [48,49,50,51]. The presence of higher densities of intermediate hosts has already been linked to abundance of parasite communities [52]; therefore, we suggest that the higher prevalence of this parasite at higher altitudes could probably be connected to a higher presence of earthworms (*Lumbricus* spp. and *Allolobophora* spp.), which act as intermediate hosts.

Moreover, infection of earthworms with *E. garfiai* could also be higher in areas located above 900 m asl due to the presence of a higher number of wild boars in these areas and the positive correlation of final host abundance to parasitic infection of intermediate hosts [53]. The association between *Metastrongylus* spp. larval infection of earthworms and wild boar density was previously demonstrated by Nagy et al. [54], and further studies could also demonstrate the validity of this theory for *E. garfiai*.

## 5. Conclusions

Soil quality and sustainability can be evaluated by using its micro and macrofauna. In particular, earthworms have been proven to be valuable bioindicators and biomonitors due to the abundance and variety of species composition of the earthworm fauna, the behavior of these invertebrates in contact with the soil substrate, and the accumulation of chemicals from the environment into their bodies [55,56,57,58,59]. Soil bacteria and parasites have also frequently been used to assess the quality of soil and of the wider ecosystems connected to it [60,61,62,63]. The present study shows a higher prevalence of *E. garfiai* in tongues of wild boars collected at higher altitudes where a higher presence of earthworms is found. Therefore, it could be useful for the evaluation of the investigated environments to add to routine tests for the identification of zoonotic parasites (*Trichinella* spp.) the assessment of *E. garfiai* occurrence in samples of tongues from wild boars hunted during the hunting season, as an indicator of earthworm presence and consequently of soil quality. However, significant research gaps still exist, and more studies should be carried out to understand the variations in parasite abundance and the possible use of *E. garfiai* as a bioindicator.

## Figures and Tables

**Table 1:** Sampling sites located below 900 m asl: sampling municipality/province, altitude, geographical coordinates, and acronyms used in the study are reported.

Municipality/Province Group < 900 m asl	Altitude Latitude N Longitude E	Acronym	Number of Samples
San Lupo (Benevento)	511 41°27′69.15″ 14°63′36.52″	SL BN < 900	5
Castel Franci (Avellino)	580	CF AV < 900	26
	40°55′16.16″ 15°3′25.83″		
San Gregorio Magno (Salerno)	547	SGM SA < 900	5
	40°40′24.20″ 15°24′41.92″		
Lenola/Taverna (Latina)	507	Ta LT < 900	7
	41°23′39.41″		
	13°30′53.01″		
Total < 900 m asl			43

**Table 2:** Sampling sites located above 900 m asl: sampling municipality/province, altitude, geographical coordinates and acronyms used in the study are reported.

Municipality/Province Group > 900 m asl	Altitude Latitude N Longitude E	Acronym	Number of Samples
Ponte Landolfo (Benevento)	1017 41°29′47.57″ 14°67′99.50″	PL BN > 900	7
Nusco (Avellino)	914	Nu AV > 900	8
	40°89′76.30″ 15°05′57.13″		
Montesano s/Marcellana (Salerno)	920	MsM SA > 900	5
	40°16′17.19″ 15°42′32.37″		
Lenola/Camposerianni (Latina)	901	Ca LT > 900	6
	41°21′39.13″		
	13°29′38.26″		
Total > 900 m asl			26

**Table 3:** Percentages of positive samples according to sampling area.

Group > 900 m asl	Positive	Group < 900 m asl	Positive
PL BN > 900	80.00%	SL BN < 900	40.00%
Nu AV > 900	62.50%	CF AV < 900	44.00%
MsM SA > 900	60.00%	SGM SA < 900	20.00%
Ca LT > 900	66.67%	Ta LT < 900	28.57%
Tot Group > 900	66.67%	Tot Group < 900	38.09%

Percentages of positivity for each sampling area and total positivity percentages for each altitude group are presented. Significance for positivity among sampling areas of the same province was *p* < 0.01.

## Data Availability

The data that support the findings of this study are available from the corresponding author upon reasonable request.

## Supplementary Materials

The following supporting information can be downloaded at: https://www.mdpi.com/article/10.3390/ani13040706/s1, Table S1: Eucoleus garfiai in wild boars in Campania and Latium regions (Italy).
