# Supplementary material for: Prevalence of Eucoleus garfiai in Wild Boars Hunted at Different Altitudes in the Campania and Latium Regions (Italy)
Source: Animals (Basel). 2023 Feb 17;13(4):706. doi: 10.3390/ani13040706 (PMC9952325; doi:10.3390/ani13040706)
Supplement: Supplementary file 1 [file animals-13-00706-s001.zip › animals-2188082-supplementary.pdf]

Table S1. ***Eucoleus garfiai*** **in wild boars in Campania and Latium regions (Italy).**

Sampling Site	Sample Number	Presence of adult/egg
SL BN <900	1	+
	2	+
	3	-
	4	-
	5	-
CF AV <900	6	+
	7	+
	8	+
	9	+
	10	+
	11	+
	12	+
	13	+
	14	+
	15	+
	16	+
	17	-
	18	-
	19	-
	20	-
	21	-
	22	-
	23	-
	24	-
	25	-
	26	-
	27	-
	28	-
	29	-
	30	-
	31	n.a.*
SGM SA <900	32	+
	33	-
	34	-
	35	-
	36	-
Ta LT<900	37	+
	38	+
	39	-
	40	-
	41	-
	42	-
	43	-

PSL BN >900	44	+
	45	+
	46	+
	47	+
	48	-
	49	n.a.*
	50	n.a.*
NuCF AV >900	51	+
	52	+
	53	+
	54	+
	55	+
	56	-
	57	-
	58	-
MsM SA >900	59	+
	60	+
	61	+
	62	-
	63	-
Ca LT >900	64	+
	65	+
	66	+
	67	+
	68	-
	69	-

Acronym of sampling sites, number of sample and positivity (+) or negativity (-) are reported. *n.a. not available
